# The EQ-5D (Euroqol) is a valid generic instrument for measuring quality of life in patients with dyspepsia

**DOI:** 10.1186/1471-230X-9-20

**Published:** 2009-03-12

**Authors:** Sanjiv Mahadeva, Hwee-Lin Wee, Khean-Lee Goh, Julian Thumboo

**Affiliations:** 1 Division of Gastroenterology, Department of Medicine, Faculty of Medicine, University of Malaya, Kuala Lumpur, Malaysia; 2 Department of Rheumatology and Immunology, Singapore General Hospital, Singapore; 3 Department of Medicine, National University of Singapore, Singapore

## Abstract

**Background:**

There is little information of the validity of generic instruments in measuring health-related quality of life (HRQOL) in patients with dyspepsia. We aimed to assess the reliability and validity of the EQ-5D, a brief and simple instrument, in measuring HRQOL in adult patients with dyspepsia.

**Methods:**

Consecutive adults with dyspepsia attending the Gastroenterology clinic in a tertiary referral center were interviewed with the EQ-5D (both English and Malay versions), the short-form Nepean Dyspepsia Index (SF-NDI), the SF-36 and Leeds Dyspepsia Questionnaire (LDQ). Known-groups and convergent construct validity were investigated by testing hypotheses at attribute and overall levels. A repeat telephone interview was conducted 2 weeks later to assess test-retest reliability.

**Results:**

A total of 113 patients (mean (SD) age: 53.7 (14) years; 49.5% male; 24.8% Malays, 37.2% Chinese; 70.8% functional dyspepsia) were recruited. Response rate was 100% with nil missing data. Known-groups validation revealed 20/26 hypotheses fulfillment. Patients with more severe dyspepsia reported more problems with their usual activity (p = 0.07) and pain (p = 0.06) and demonstrated lower median VAS scores (60 vs 70, p = 0.002) and EQ-5D utility scores (0.72 vs 0.78, p = 0.002). Those reporting problems in various EQ-5D dimensions had significantly lower scores in relevant SF-36 and SF-NDI dimensions. The overall EQ-5D utility score also demonstrated good correlation with the SF-36 summary physical and mental scores and the SF-NDI total score. Intraclass correlation coefficient for test-retest reliability was 0.66 (95% CI = 0.55 – 0.76).

**Conclusion:**

The EQ-5D is an acceptable, valid and reliable generic instrument for measuring HRQOL in adult patients with dyspepsia.

## Background

Dyspepsia, a collection of upper gastrointestinal symptoms due to various causes, is a common problem worldwide [1]. As most patients with dyspepsia have functional disease, the treatment of which remains unsatisfactory at present [2], health-related quality of life (HRQOL) measurement has become an important clinical objective in the assessment of new therapies for this condition [3]. Several disease-specific HRQOL instruments for dyspepsia have been developed including the quality of life in reflux and dyspepsia (QOLRAD) questionnaire [4] and the Nepean Dyspepsia Index [5], both of which are valid and reliable tools for assessing HRQOL in patients with dyspepsia. These instruments are more responsive to change in HRQOL in dyspeptic patients, especially following therapy, and hence ideal for evaluating newer medical therapies, etc.

However, disease-specific instruments for dyspepsia do not allow for comparisons with patients with illnesses other than dyspepsia and with healthy individuals in the general population. Generic HRQOL instruments are therefore valuable to supplement disease-specific instruments to enable such comparisons. The Short Form 36 Health Survey (SF-36) is one of the most widely accepted generic instruments for measuring HRQOL in many countries [6]. However, it is relatively long and may not be appropriate for large population surveys involving direct interview techniques. The EQ-5D is another generic HRQOL instrument that has been shown to be valid and reliable in the general population and various patient groups [7-9]. It is brief, comprising of five questions and a visual analogue scale, which makes it easy to use and particularly attractive in large scale population surveys, for surveys in the elderly and those with lower educational levels. It has the additional advantage of allowing incorporation of HRQOL data into pharmacoeconomic analyses.

To compare HRQOL of dyspeptic patients with those of the general population, the generic instrument has to be shown to be valid and reliable in dyspepsia first. To our knowledge, the EQ-5D has not been validated for this purpose before and we therefore aimed to examine this in a group of Malaysian patients with dyspepsia attending regular follow up at a tertiary institution.

## Methods

### Subjects

Ethical approval was obtained by the University Malaya Medical Centre Ethics Committee prior to the commencement of this study. Consecutive adult outpatients with dyspepsia attending the Gastroenterology clinic of this tertiary teaching institution were invited to participate in the study. Most of these patients had functional or non-ulcer dyspepsia (defined as dyspepsia with a normal or minor endoscopic findings), with the remaining attending follow up for organic disease (endoscopic findings of duodenal erosions, peptic ulcer disease and erosive oesophagitis). Patients were interviewed by a trained research assistant using HRQOL and disease severity instruments validated for use in Malaysia (see below) in English or Malay (depending on individual patient's language proficiency). Additional information obtained included period of medical consultation and socio-economic-demographic status. Test-retest reliability of EQ-5D was evaluated by administering the EQ-5D twice to the same subjects, 2-weeks apart, and comparing the scores obtained on these two occasions. The second interview was conducted over the telephone by our trained research assistant.

### Instruments

*The EQ-5D *comprises five questions on mobility, self-care, pain, usual activities and psychological status with three possible answers for each item (1 = no problems, 2 = moderate problems, 3 = severe problems) [7]. An overall utility score is calculated based on these domains, with a range score from 0 (worse health scenario) to a maximum of 1.0 (best health scenario). An additional visual analogue scale (VAS, scale 0 – 100) is used to assess general health status with 100 indicating the best health status. Malaysian English and Malaysian Malay versions of the EQ-5D were developed by the EuroQoL Group: 2005 (original developers) using their standard translation and linguistic validation process [10] and have been validated for use in Malaysia.

*The SF-36 *is an established generic HRQOL instrument which comprises 36 questions and eight different sub-scores: physical functioning, physical role limitations, bodily pain, general health perceptions, vitality, social functioning, emotional role limitations, mental health and 2 composite scores – Physical Component (PCS) and Mental Component Scores (MCS) scored on a 0 to 100 scale [11]. The maximum score of 100 indicates the best possible health state. English and Malay versions have previously been validated in Malaysia and shown to be a reliable measure of general QOL health status [12].

The *Short Form (SF) Nepean Dyspepsia Index *is a disease-specific HRQOL instrument for dyspepsia, comprising a 10-item questionnaire examining the influence of dyspepsia on five elements (sub-scales) of health in patients, namely tension/anxiety, interference with daily activities, disruption to regular eating/drinking, knowledge towards/control over disease symptoms and interference with work/study [13]. Each item is measured by a 5-point Likert scale ranging from 0 (not at all or not applicable), 1 (a little), 2 (moderately), 3 (quite a lot) to 4 (extremely). Individual items are subsequently computed to obtain a score range from 0 (lowest HRQOL score) to 100 (highest HRQOL score) as per the developers' original calculation formula [5]. A total, overall SF-NDI score is obtained by the summation of all sub-scale scores followed by division of five. This instrument has previously been shown to be valid, sensitive and reliable for measuring HRQOL status in Malaysian patients with dyspepsia [14]

The *Leeds Dyspepsia Questionnaire *(LDQ), is an eight item symptom-based questionnaire relating to the frequency and severity of various upper G.I. symptoms, namely upper abdominal pain/discomfort, heartburn, regurgitation, dysphagia, belching, nausea, vomiting and post-prandial distension/early satiety [15]. The total score ranges from 0 – 40, with lower values indicating less severity and higher values more severe dyspepsia. A score of 15 or more had been defined by the developers as indicative of severe dyspepsia. The questionnaire has previously been validated in our local population and shown to be reliable in assessing dyspepsia amongst Malaysians [16].

### Psychometric evaluation

Psychometric properties of the EQ-5D were evaluated by assessing its frequency of missing data, ceiling and floor effects (i.e. percentage of samples achieving highest and lowest values), test-retest reliability and construct validity. The reliability of an instrument refers to its ability to yield reproducible and consistent results. Construct validity is an assessment of the degree to which an instrument measures the construct that it was designed to measure. The process involves forming hypothetical models to describe the constructs being assessed and postulating their relationship. An assessment of the relationship between the construct data is then made to confirm or refute prior expectations and hence determine the validity of the instrument being tested [17].

Construct validity of the EQ-5D was assessed by testing 26 a-priori hypotheses related to item or utility scores as follows: a) patients with less favourable demography in relation to dyspepsia would report more problems with relevant EQ-5D dimensions, b) patients with more severe dyspeptic symptoms would have more problems with relevant EQ-5D dimensions and lower utility scores c) subjects reporting problems for any EQ-5D dimensions would have lower scores for relevant SF-36 and SF-NDI scales and d) utility scores for EQ-5D would correlate well with SF-36 and SF-NDI summary scores. Test-retest reliability of EQ-5D was evaluated by administering the EQ-5D twice to the same subjects, 2-weeks apart, and comparing the scores obtained on these two occasions. The first interview was conducted face-to-face and the second interview was conducted over the telephone, both by our trained research assistant.

### Statistics

Hypothesized trends were tested using Chi-square or Mann-Whitney tests, or Spearmans' correlation coefficient where appropriate. Strong, moderate and weak correlations were defined as > 0.60, 0.30 – 0.60 and < 0.30 respectively [18]. Test-retest reliability of the EQ-5D utility score was assessed using intraclass correlation coefficients, with a desired value of > 0.6 [17]. Statistical significance for hypothesis fulfillment was defined as a p value of < 0.05. Data were analysed with SPSS for windows (version 12, SPSS Inc, IL, USA).

## Results

### Patient characteristics

A total of 113 consecutive patients with dyspepsia were interviewed between May and October 2006 and their characteristics and basic demography are summarized in Table 1. The mean age of patients was 53.7 ± 14 years, the male:female ratio was 1:1.02 and their ethnicity were as follows: 28 (24.8%) Malays, 42 (37.2%) Chinese and 41 (36.3%) Indians. 90.3% of patients had above secondary level education and most patients (41.6%) were retired at the time of interview. The majority of cases (70.8%) were functional dyspepsia and the median period of medical consultation (either primary care or hospital specialist) was 6 years (range 3 – 18). The median LDQ score in patients was 16 (11 – 22), indicating that most patients had fairly severe or poorly-controlled symptoms despite many years of treatment.

**Table 1:** Characteristics and demography of Malaysian patients with dyspepsia in the study

	n = 113
**Mean age (range)**	53.7 ± 14 (17 – 83)

**Gender (Male: Female)**	1: 1.02

**Ethnicity:**	
**Malay**	28 (24.8%)
**Chinese**	42 (37.2%)
**Indian**	41 (36.3%)

**Education level:**	
**Primary (6 years of education)**	11 (9.7%)
**Secondary (12 years of education**	68 (60.2%)
**Tertiary (at least 15 years of education)**	34 (30.1%)

**Marital status:**	
**Unmarried/separate/divorced**	21 (18.6%)
**Married**	84 (74.3%)
**Widowed**	8 (7.1%)

**Employment status:**	
**Employed**	42 (37.2%)
**Unemployed/homemaker**	24 (21.2%)
**Retired**	47 (41.6%)

**Diagnosis:**	
**Functional dyspepsia**	80 (70.8%)
**Peptic ulcer disease**	6 (5.3%)
**Gastroesophageal reflux disease**	27 (23.9%)

**Length of dyspeptic symptoms (years)**	
**(median; interquartile range)**	7.5 (3 – 20)

**Period of medical consultation (years)**	
**(median; interquartile range)**	6 (3 – 18)

**LDQ score (median; interquartile range)**	16 (11 – 22)

### EQ-5D data

**Figure 1:**
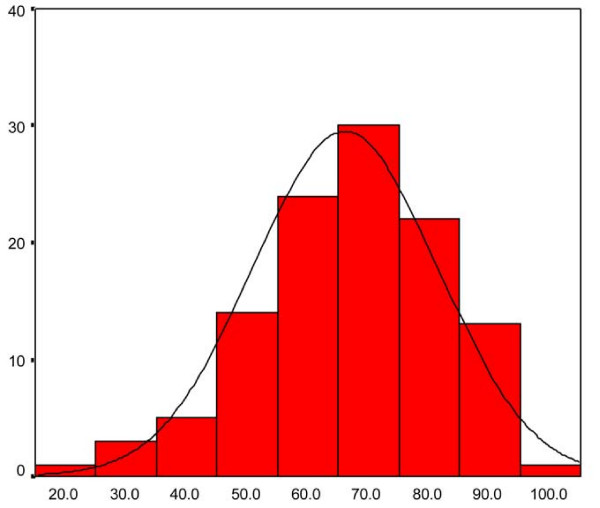
**Histogram demonstrating range and distribution of EQ-5D VAS scores**.

The response rate was 100% with no missing data for all variables. The calculated utility score had a median value of 0.725 (skewness – 1.78), with a range from as low as 0.077 to a maximum of 1.00 (best health scenario). The overall VAS had a median value of 70.0 (skewness – 0.52), with a range from 20.0 to 95.0 (Figure 1).

### Test-retest reliability

Ninety four (83.2%) patients participated in the follow-up telephone interview, which was conducted at a median of 16 days (range 13 – 18) after the original interview. Intraclass correlation coefficient for the EQ-5D utility score was 0.66 (95% CI = 0.55 – 0.76, p < 0.0001).

### Validity

Twenty of the 26 hypotheses relating EQ-5D dimensions/summary scores to other variables were fulfilled. At the attribute level, problems with usual activity and pain were associated with worsening dyspeptic symptoms but these were not statistically significant. However, significantly lower values for the EQ-5D utility scores and VAS were demonstrated with severe dyspepsia (Table 2). All hypotheses relating EQ-5D dimensions to relevant SF-36 sub-scales were supported (see Additional file 1). For example, patients who reported problems with mobility in the EQ-5D had significantly lower scores in relevant SF-36 sub-scales (i.e. physical functioning and role physical), whilst those reporting problems with anxiety had significantly lower scores for SF-36 mental health and role emotional sub-scales (see Additional file 1). Five out of the 7 hypotheses relating EQ-5D dimensions to relevant SF-NDI sub-scales were supported (see Additional file 1). In particular, patients reporting problems with usual activities and pain demonstrated significantly lower scores in the "interference" and "eating" sub-scales, whilst those reporting problems with anxiety had significantly lower scores in the "tension" sub-scales. Utility scores of the EQ-5D demonstrated good correlation with the SF-36 physical component summary score (r = 0.45), the SF-36 mental component summary score (r = 0.49) and the cumulative SF-NDI score (r = 0.47) (see Additional file 1).

**Table 2:** Relationship of EQ-5D dimensions, utility scores and VAS to other relevant variables

EQ-5D dimension/utility score vs variable	n (%)	p
**Usual activity vs severe dyspepsia #**		
**No problems**	45 (72.6%)	0.07
**Got problems**	17 (27.4%)	

**Pain vs severe dyspepsia #**		
**No pain**	10 (16.7%)	0.06
**Moderate/extreme pain**	50 (83.3%)	

**Utility score vs dyspepsia#**		
**Mild**	0.78 *	0.002
**Severe**	0.72	

**VAS scale vs dyspepsia**		
**Mild**	60*	0.002
**Severe**	70	

* median value

# Defined by LDQ – Mild < 15, Severe ≥ 15

## Discussion

The brevity and simplicity of the EQ-5D makes it particularly attractive for use in large scale population surveys of dyspepsia. Furthermore, a single utility score summarizing all 5 dimension states in health can be used to calculate quality-adjusted life years (QALYs) for cost-utility analysis of interventions in dyspepsia, where most patients have functional disease with variable outcomes. However, before the EQ-5D can be utilized for dyspepsia in this manner, it has to demonstrate satisfactory psychometric properties in this condition. In this study of patients with dyspepsia from a variety of causes, from different ethnic backgrounds, of varying ages (from 17 – 83 years) and with > 90% secondary education, the EQ-5D was found to be reliable and to have acceptable construct validity, suggesting that it could be a useful generic HRQOL measure for patients with dyspepsia.

Reliability assessment of a HRQOL instrument consists of determining that a measurement yields consistent and reproducible results [17]. We used the test-retest reliability method in our population of stable dyspeptics and demonstrated moderate to good reliability of the EQ-5D in dyspeptics. Possible discrepancy with the follow-up interview technique, as this was telephone based compared to a face-to-face method at baseline may have resulted in a lower intraclass correlation coefficient value. It was unlikely that any major change in dyspeptic symptoms could have occurred in the interval of (median) 2 weeks between interviews as most patients had a prolonged duration of symptoms (median 7.5 years, range 3 – 20 years) and were on regular anti-dyspeptic therapy.

The validity of the EQ-5D was demonstrated by the fulfillment of twenty of the 26 hypotheses based on known-groups construct validity. Hypothesized relationships for the remaining six hypotheses were present, but failed to reach statistical significance. For dyspepsia in particular, the total utility score and the dimension of "pain" were able to demonstrate poorer HRQOL outcomes in patients with more severe symptoms. As the EQ-5D derives information for each dimension of HRQOL from only one item, it is important that we demonstrate this to be sufficient by comparing it against a more detailed and established multi-dimension instrument. In this study, we demonstrated that patients reporting problems in all five dimensions of the EQ-5D had significantly lower scores in relevant dimensions of the SF-36. Furthermore, the overall EQ-5D utility score showed good correlation with the SF-36 physical and mental summary scores, further substantiating the validity of the EQ-5D.

A possible limitation of many generic HRQOL instruments are that dimensions of health being measured tend to be more physical or mobility-based. In this context, published literature on the validity of the EQ-5D in specific diseases have mainly been shown for physically limiting illnesses such as Parkinson's disease [8], rheumatological conditions [9] and stroke [19]. In this study, we compared EQ-5D dimensions to a dyspepsia-specific HRQOL instrument, the SF-NDI, and found that physical dimensions dealing with "mobility" and "self-care" had poor (i.e. non-significant or non-hypothesized trend) associations and were not relevant to dyspepsia-related HRQOL. This is not surprising at all as dyspepsia rarely, if ever, causes problems with mobility and inability to self-care. However, dyspepsia is known to be associated with psychological disorders and can result in work-absenteeism [20]. Accordingly, we demonstrated that patients reporting problems in dimensions of the EQ-5D relating to "usual activities" and "anxiety/depression" had significantly lower scores in relevant SF-NDI sub-scales. Additionally, those reporting problems with "pain/discomfort" had lower scores with most SF-NDI sub-scales (abdominal pain or discomfort being a predominant symptom in dyspepsia) and the utility score correlated well with the SF-NDI summary score.

A limitation of this cross-sectional study was the inability to assess other properties of the EQ-5D in relation to dyspepsia, such as its' responsiveness. The latter requires a cohort study to examine the ability of an instrument to detect change in HRQOL status.

## Conclusion

We are able to conclude that the EQ-5D is an acceptable, valid and reliable generic instrument for measuring health-related HRQOL in adult patients with dyspepsia. Our data are consistent with other studies exploring the validity of the EQ-5D in various disease states [8,9,19], although most of these have been either neurological or rheumatological conditions. We believe that the EQ-5D may be useful in measuring health-related HRQOL in other gastroenterological diseases, although further validation studies will have to be conducted to determine this.

## Competing interests

The authors declare that they have no competing interests.

## Authors' contributions

SM and HLW designed the study, analysed and interpreted the data, and drafted the manuscript. KLG provided administrative support and contributed to data collection with SM. JT provided technical support and critical revision of the manuscript. All authors reviewed and approved final version of the manuscript.

## Pre-publication history

The pre-publication history for this paper can be accessed here:

http://www.biomedcentral.com/1471-230X/9/20/prepub

## Supplementary Material

Additional file 1 **Median scores and correlations of SF-36 (generic) and SF-NDI (disease-specific) dimensions with those of various EQ-5D domains. **The data provided represents correlation analysis between HRQOL domains of the SF-36 and SF-NDI against the EQ-5D domains, whereby similarities in relevant domains of HRQOL are highlighted. Click here for file
