# Supplementary material for: The EQ-5D (Euroqol) is a valid generic instrument for measuring quality of life in patients with dyspepsia
Source: BMC Gastroenterol. 2009 Mar 12;9:20. doi: 10.1186/1471-230X-9-20 (PMC2662871; doi:10.1186/1471-230X-9-20)
Supplement: Additional file 1 — Median scores and correlations of SF-36 (generic) and SF-NDI (disease-specific) dimensions with those of various EQ-5D domains. The data provided represents correlation analysis between HRQOL domains of the SF-36 and SF-NDI against the EQ-5D domains, whereby similarities in relevant domains of HRQOL are highlighted. [file 1471-230X-9-20-S1.doc]

**Table 3** - Median scores and correlations of SF-36 (generic) and SF-NDI (disease-specific) dimensions with those of various EQ-5D domains

|  | SF-36 | | | | | | | | | | SF-NDI | | | | | |
| --- | --- | --- | --- | --- | --- | --- | --- | --- | --- | --- | --- | --- | --- | --- | --- | --- |
|  | PF | RP | BP | GH | VT | SF | RE | MH | PCS | MCS | Interfere | Eating | Know-ledge | Work/  study | Tension | Total score |
| EQ-5D |  |  |  |  |  |  |  |  |  |  |  |  |  |  |  |  |
| Mobility  No problem  With problems | 80** | 68.8** | 62 | 60 | 62.5 | 75 | 75 | 70 |  |  | 33.3 | 33.3 | 33.3 | 33.3 | 30.6 |  |
|  | 60 | 50 | 31 | 50 | 50 | 50 | 50 | 50 |  |  | 33.3 | 27.8 | 27.8 | 33.3 | 27.8 |  |
| Self-care  No problem  With problems | 80 | 62.5* | 52 | 57 | 56.2 | 75* | 75 | 65 |  |  | 33.3 | 33.3 | 33.3 | 33.3 | 27.8 |  |
|  | 40 | 31.2 | 26.5 | 46 | 40.6 | 37.5 | 29.2 | 47.5 |  |  | 27.8 | 30.6 | 27.8 | 27.8 | 30.6 |  |
| Usual activities  No problem  With problems | 80 | 75** | 62 | 60** | 62.5** | 75 | 75 | 70 |  |  | 33.3 | 33.3* | 33.3 | 33.3* | 33.3 |  |
|  | 67.5 | 50 | 41.5 | 46 | 47.5 | 50 | 50 | 57.5 |  |  | 27.8 | 19.4 | 33.3 | 27.8 | 25.0 |  |
| Pain/ discomfort  No pain  With pain | 80 | 75 | 62.0** | 62 | 68.8 | 75 | 83.3 | 70 |  |  | 38.9** | 33.3** | 33.3 | 33.3 | 38.9** |  |
|  | 75 | 62.5 | 51.0 | 52 | 56.3 | 62.5 | 58.3 | 65 |  |  | 33.3 | 27.8 | 33.3 | 33.3 | 27.8 |  |
| Anxiety/ depression  No problems  With problems | 80 | 72 | 62 | 62 | 62.5 | 81.3 | 75** | 80** |  |  | 38.9 | 33.3 | 33.3 | 33.3 | 33.3* |  |
|  | 75 | 56.3 | 51 | 52 | 50 | 62.5 | 58.3 | 65 |  |  | 33.3 | 33.3 | 33.3 | 33.3 | 27.8 |  |
| Utility score |  |  |  |  |  |  |  |  | (0.45)** | (0.49)** |  |  |  |  |  | (0.47)** |

PF-physical functioning; RP-role physical; BP-bodily pain; GH-general health; VT-vitality; SF-social functioning; RE-role emotional; MH-mental health; PCS-physical component score; MCS-mental component score

* p < 0.05; ** p < 0.01, ( ) – correlation coefficient values

**NB : Shaded boxes refer to relevant domains for comparison between the 3 HRQOL instruments**
